# Bifocal osteosynthesis to treat radial shortening deformity with dislocation of the inferior radioulnar joint

**DOI:** 10.1186/s12891-019-2816-5

**Published:** 2019-10-10

**Authors:** Junjie Guan, Hongjiang Ruan, Jimin Yin, Yimin Chai, Qinglin Kang

**Affiliations:** Department of Orthopedics surgery, Shanghai Jiao Tong University Affiliated Sixth People’s Hospital, Number 600, Yishan Road, Xuhui district, Shanghai, China

**Keywords:** Radial shortening deformity, Bifocal osteosynthesis, Limb lengthening, Correction

## Abstract

**Background:**

Several methods have been reported to correct deformity and shortening of the distal radius. However, the results are not entirely satisfactory. The results of bifocal osteosynthesis were retrospectively analyzed in this study.

**Methods:**

Eight patients treated with bifocal osteosynthesis were evaluated retrospectively. Pre-operative and post-operative clinical and radiographic examinations were performed. Subjective symptoms and objective joint function were assessed. Radiographic data of the extent of radial lengthening and distal radial articular angle were collected.

**Results:**

The mean follow-up period was 46 months (37–68 months). Satisfactory wrist appearance and radial lengthening was achieved in all patients. All patients were satisfied with the wrist appearance and willing to undergo the same treatment again. The range of motion (ROM) of the forearm and wrist was significantly improved. Pin-track infections occurred in two patients, for which they received wound care and oral antibiotics. Complications such as fixation device failure, tendon rupture, fracture of regenerated bone or nerve impairment did not occur. The duration of lengthening depended on the shortening of the radius. Delayed union in the docking site was observed in two patients and union was achieved after bone grafting.

**Conclusions:**

Bifocal osteosynthesis using the Ilizarov method provides a useful method for correction of radial shortening deformity with dislocation of the inferior radioulnar joint. Despite the fact that we did not validate pre-and post-operation functional outcome scores, all patients were satisfied with the wrist appearance and function.

## Background

Radial length discrepancies due to exostosis, trauma to the epiphyseal plate or congenital deficiencies will result in functional and cosmetic problems [1, 2]. Radial length discrepancies not only result in limb shortening but are also accompanied by wrist dysfunction and dislocation of the inferior radioulnar joint [3, 4]. Treatment for radial lengthening deformities is complicated. The aim of surgical treatments is to restore function and relieve pain. Although different methods have been reported to correct the deformity, the results of those treatments are not entirely satisfactory [5, 6]. Generally, the restoration of radial length, volar and ulnar inclination and congruity of the wrist joint are key in achieving a successful outcome.

**Fig. 1:**
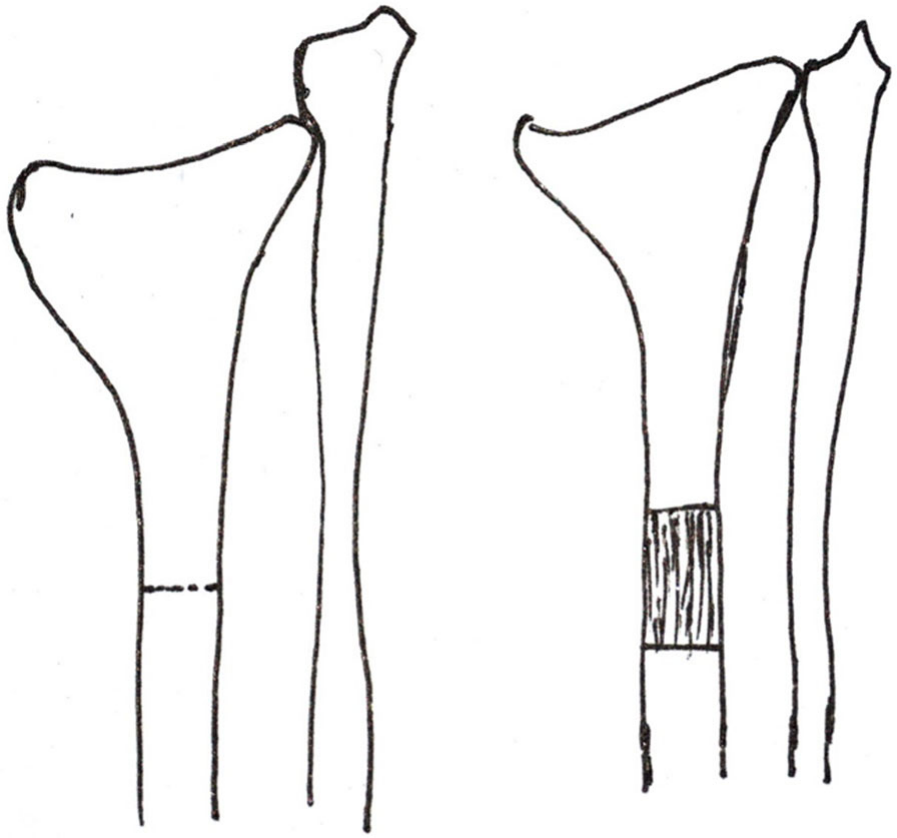
Osteotomy and lengthening in the radial shaft

**Fig. 2:**
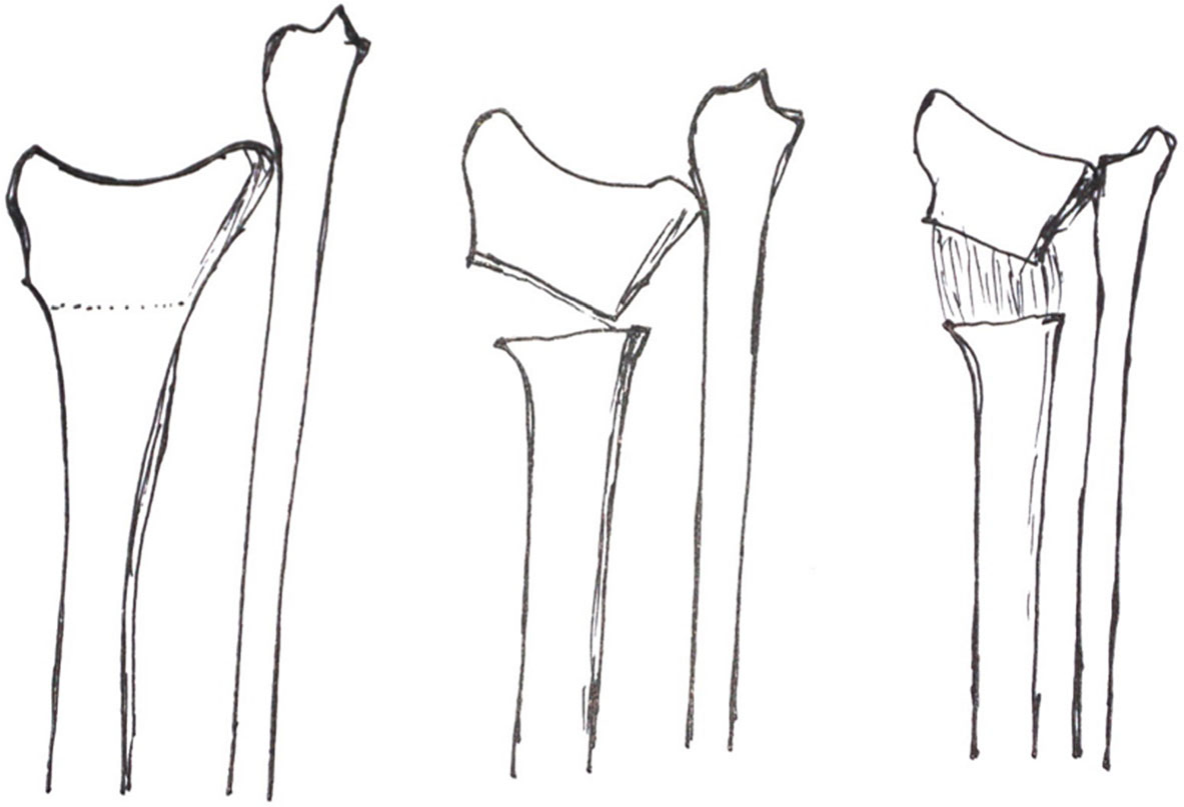
Osteotomy, correction and lengthening in the distal radial

Ilizarov explained in detail the concept of “distraction-induced osteogenesis”, demonstrating that the application of slow, steady traction activates bone regeneration [5]. The design of external fixation devices and advances in our understanding of the biological response to distraction has pushed forward the clinical application of “distraction-induced osteogenesis” [7]. However, the best way to apply the Ilizarov technique to solve the problem of distal radial deformity and shortening is still controversial [1]. Simple radial shaft lengthening only solves the problem of radial shortening, but cannot restore volar tilt, ulnar inclination or the congruence of the wrist joint (Fig. 1) [8]. The standard method involves osteotomy of the distal radius, which lengthens the bone and restores volar tilt and ulnar inclination. However, the lengthening of the distal radius is often unsatisfactory (Fig. 2). Meanwhile, it is difficult to maintain volar tilt and ulnar inclination through external fixation during the process of distraction.

**Fig. 3:**
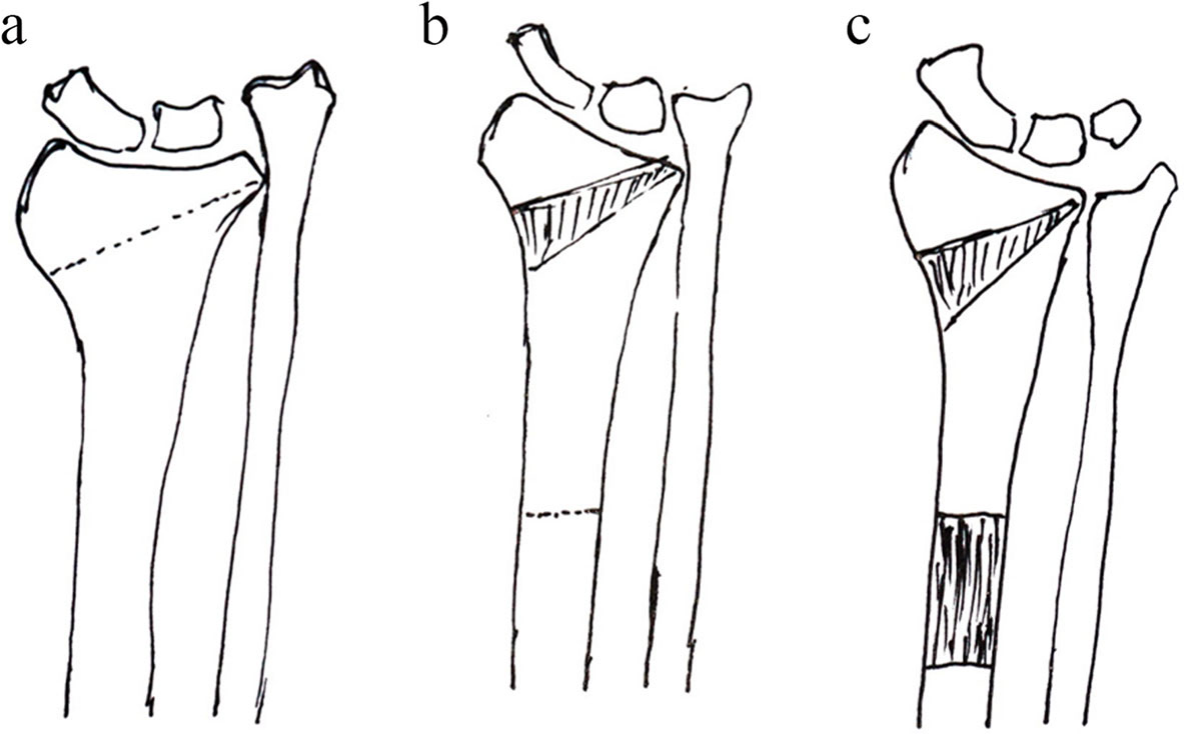
**a** Osteotomy for distal radial correction. **b** Osteotomy for the radial lengthening. **c** The final lengthening and correction effect

In this study, we describe our technique using bifocal osteosynthesis to treat distal radial deformity and shortening (Fig. 3). Pre-operative and post-operative clinical and radiologic parameters were collected. The outcome was evaluated over a medium-term follow-up period. The results showed that the adoption of bifocal osteosynthesis was useful for the radial shortening deformity.

## Methods

Eight patients (5 males and 3 females) with distal radius deformity and radial shortening who underwent bifocal osteosynthesis were included in the current retrospective analysis. Patient ages ranged from 25 to 38 years at the time of lengthening (mean age = 30.75 years, standard deviation = 4.77). All had suffered from distal radial growth plate injury.

### Surgical procedure

The same senior orthopedic surgeon (Qinglin Kang) performed all surgeries. The patients were operated on in a supine position under general anesthesia. The forearm was placed on a radiolucent table. The bifocal osteosynthesis involved osteotomy of the distal radius and the radial shaft. To restore congruity of the wrist, osteotomy of the distal radius was performed under fluoroscopy. When the desired volar tilt and ulnar inclination was achieved, an iliac bone graft was inserted into the wedge osteotomy site. Two cannulated screws were placed to maintain the correction. For the distraction osteotomy of the radius, two pins were inserted proximal to the osteotomy site. The external fixator was applied to the proximal pins. The distal pins were positioned using the external fixator as a guide. The threads of the pins were completely buried to prevent tendon injury or rupture. The external fixator was removed and a tiny incision was made in the radius shaft. A hand surgery oscillating saw was used to create the corticotomy. The external fixator was re-inserted and fastened to the pins. A distraction test was performed to make sure that the osteotomy was complete. Daily lengthening was started at a rate of 0.25 mm three times per day (at 07:00, 14:00, and 21:00), commencing 7 days after operation. Swelling of the hand and the quality of the distracted bone were monitored. After sufficient radial length was obtained, distraction was terminated. The fixator was left in place until consolidation, as determined via X-ray. Active and passive exercises of the fingers, wrist and elbow were encouraged throughout the treatment period.

## Results

**Fig. 4:**
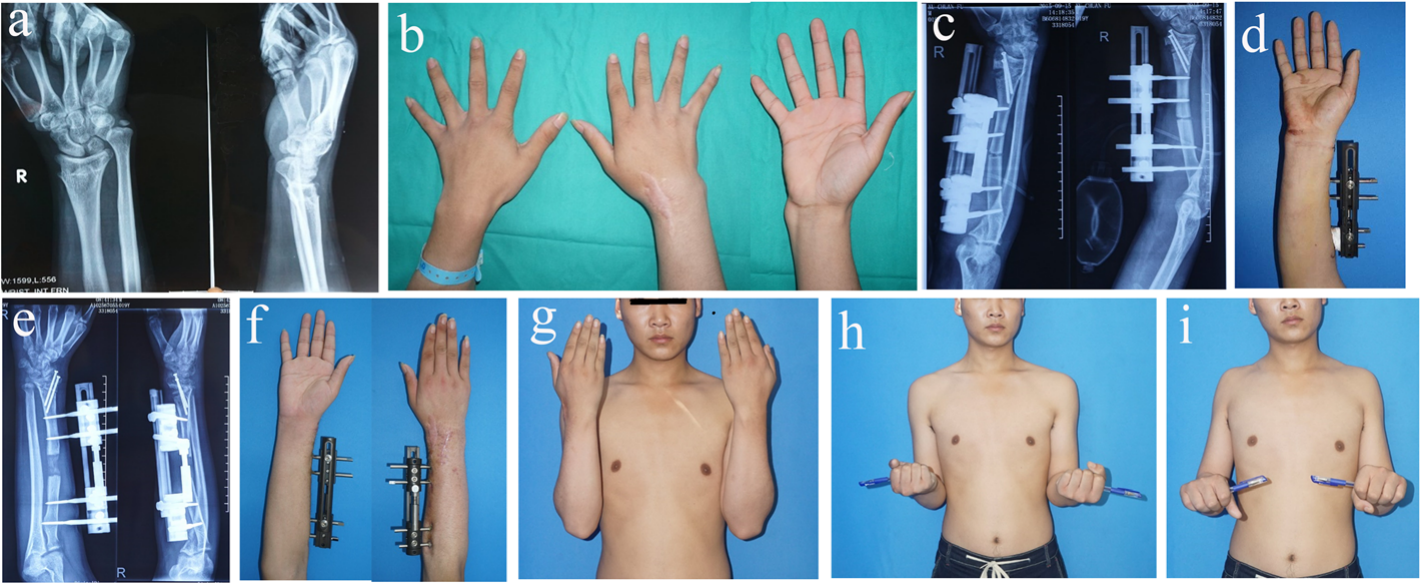
**a**, **b** A patient suffered radial shortening with distal ulnar-radial dislocation. **c**, **d** Postoperative X-ray. **e**, **f** X-ray and gross observation when distraction was terminated. **g**, **h** The cosmetic and functional results

Patients were followed up for an average of 46 months (range: 37 to 68). At the time of final follow-up, all patients had achieved osseous union and were satisfied with the functional and cosmetic results (Fig. 4).

The average volar tilt was − 14 (range: − 20 to − 8, standard deviation = 4.4) preoperatively and 16 (range: 10 to 20, standard deviation = 3.4) postoperatively. The average ulnar inclination increased from − 5 (range: − 10 to 3, standard deviation = 4.0) preoperatively to 14 (range: 10 to 18, standard deviation = 3.4) postoperatively. The average lengthening achieved was 36 mm (range: 32 to 42 mm, standard deviation = 0.36). The mean distraction period was 45 days (range: 32 to 54 days, standard deviation = 7.7). The average consolidation period was 11 weeks (7 to 15 weeks, standard deviation = 2.4). The mean period of fixator treatment was 7.6 weeks (6.3–9.2 weeks, standard deviation = 1.1). The mean bone-healing index was 38 days/cm (range 34–46 days/cm, standard deviation = 0.47). Demographic and treatment results of the patients are summarized in Table 1. Two patients developed pin-site infections. All infections were successfully treated with wound care and oral antibiotics. Neither neurovascular injuries nor muscle contracture problems were observed. Delayed union at the docking site was observed in two patients after three months. Autogenous bone grafting was performed and subsequent union was achieved in both patients. The mean VAS score was 6 (range 4–8, standard deviation = 1.2) preoperatively and 2 (range 1–4, standard deviation = 1.0) postoperatively, which has a significant statistical difference (*P* < 0.05).

**Table 1:** Details of the 8 patients

		Angle of volar tilt/ulnar inclination(°)			
Case	Age-range (yrs)	Pre-operative	At latest follow-up	Lengthening (cm)	Healing index (days/cm)
1	20–30	−16/3	14/18	3.4	3.5
2	20–30	−18/−3	16/14	3.8	3.3
3	30–40	−14/− 8	10/12	3.3	3.4
4	30–40	−8/−3	20/14	3.5	3.8
5	20–30	−16/−10	14/10	4.0	4.6
6	20–30	−8/−6	20/12	3.4	4.4
7	30–40	− 12/− 5	18/20	3.2	3.6
8	20–30	20/−8	16/12	4.2	3.8

## Discussion

Radial shortening deformity with dislocation of the inferior radioulnar joint leads to functional impairment and cosmetic problems. To date, a number of surgical procedures have been advocated to treat this impairment [9–11]. Mader et al. treated 10 forearm deformities by callus distraction using a monoliteral external fixator after osteotomy. Target length was achieved in all cases. However, the wrist joint anatomy is hard to corretct [9]. Two-stage distraction lengthening of the forearm has been reported. The complication rates are high, and the overall process is arduous and prolonged [10]. Sammer et al. reported that distal radius malunions was corrected using internal osteotomy and distraction device. Even though statistically significant improvements were made, all patients had substantial residual disability [11]. There is still no general agreement on the most suitable treatment of the radial shortening deformity [12]. One of the main difficulties is restoring the radius length and congruity of the wrist joint.

The osteotomy site is a crucial part of bone lengthening. For radial lengthening, different osteotomy sites have been used in the clinical setting. Distal radial osteotomies are the most frequently used procedure for radial shortening deformity. However, the lengthening that can be obtained using distal radial osteotomies is limited. It is plausible that lengthening may be much more easily achieved in the middle of the radius. Radial shortening deformity not only causes a length discrepancy, but also results in deformity of the distal radius and incongruity of the wrist joint. To correct the distal radial deformity and the incongruity of the wrist joint, the volar tilt and ulnar inclination of the distal radius must be realigned. We used bifocal osteosynthesis to correct the deformity of the distal radius. The outcomes in our patients proved satisfactory.

For forearm lengthening, special attention should be paid to the high frequency of complications. Previous reports in the literature have documented complication rates of forearm lengthening as high as 75 to 100%. Pin infections, skin irritation and nonunion are the main distractor-related complications. The complication frequency in our study was much lower. A better understanding of the biology of distraction osteogenesis contributed to improved healing and better function. Subperiosteal osteotomy was used to preserve the periosteal sleeve. This method can protect the blood supply as much as possible. Distraction began 7 to 10 days after osteotomy. Previous reports demonstrated that delayed distraction permits the endosteal vessels to reconstitute. In addition, the nerves, vessels, and muscle are well able to tolerate a slow distraction of 0.75 mm/d [13].

The external fixator did not span the wrist joint. Throughout the distraction period, patients were encouraged to exercise their wrist and fingers, which prevented wrist and finger stiffness.

The limitations of our study are that it is a retrospective study and there was a selection bias. Another potential limitation is that there was no control group to compare the different techniques used for radial shortening deformity. However, our study showed that the functional and cosmetic outcomes were improved in all patients. Future studies are required to assess the functional outcomes such as grip strength and ROM of the forearm and wrist.

## Conclusion

In summary, for the treatment of radial shortening deformity with dislocation of the inferior radioulnar joint, biofocal osteosynthesis can achieve excellent functional and cosmetic results.

## Data Availability

The datasets used and analysed in the current study are available from the corresponding author on reasonable request.

## Abbreviation

ROM: Range of motion
